# Human serum albumin modified in myeloperoxidase-dependent reactions is a mediator of neutrophil extracellular trap formation

**DOI:** 10.7555/JBR.39.20250069

**Published:** 2026-03-19

**Authors:** Daria V. Grigorieva, Nikolay P. Gorbunov, Valeria A. Kostevich, Alexey V. Sokolov, Liliya Yu. Basyreva, Ekaterina V. Shmeleva, Tatyana V. Vakhrusheva, Sergey A. Gusev, Irina V. Gorudko, Oleg M. Panasenko

**Affiliations:** 1 Department of Biophysics, Faculty of Physics, Belarusian State University, Minsk 220030, Belarus; 2 Saint-Petersburg Pasteur Institute, Saint-Petersburg 197101, Russia; 3 Department of Biophysics, Lopukhin Federal Research and Clinical Center of Physical-Chemical Medicine of Federal Medical-Biological Agency, Moscow 119435, Russia; 4 Department of Molecular Biology of Viruses, Smorodintsev Research Institute of Influenza, Saint-Petersburg 197022, Russia; 5 Department of Medical Biophysics, Pirogov Russian National Research Medical University, Moscow 117997, Russia

**Keywords:** NETosis, neutrophil extracellular traps, human serum albumin, myeloperoxidase, hypochlorous acid, reactive halogen species

## Abstract

Activation of neutrophil membrane receptors initiates intracellular signal transduction cascades that orchestrate the cell's effector functions, including phagocytosis, production of reactive oxygen and halogen species, degranulation, and NETosis (formation of neutrophil extracellular traps [NETs]). NETs, which contain antimicrobial compounds such as myeloperoxidase (MPO), represent a strategy to combat infection. However, excessive production of NETs promotes thrombosis, diabetes mellitus, and other diseases. Therefore, investigations into the mechanisms of NETosis and the identification of modulators of this process are critical for developing strategies to address NETosis-related disorders. Here, we identified a novel NETosis inducer, human serum albumin (HSA) modified by the MPO product hypochlorous acid (HSA_HOCl_), whose accumulation *in vivo* was correlated with inflammatory processes. Using human blood neutrophils, we investigated HSA_HOCl_-induced NETosis and detected NET formation by flow cytometry. The results showed that the mechanism of HSA_HOCl_-induced NETosis involved MPO, NADPH oxidase, and phosphatidylinositol 3-kinases (PI3Ks), and that HSA_HOCl_ activated a reactive oxygen species-dependent suicidal type of NETosis. Moreover, HSA_HOCl_-induced NETosis was inhibited by an anti-HSA_HOCl_ monoclonal antibody. Thus, our findings may facilitate the development of strategies to modulate NETosis in inflammation associated with elevated MPO activity.

## Introduction

Superoxide anion radical (О_2_^•−^), which is generated *via* neutrophil NADPH oxidase (EC 1.6.99.6), is a precursor of other oxidants such as reactive oxygen species (ROS) (•OH, Н_2_О_2_, *etc.*), reactive nitrogen species (RNS) (ONOO^−^, *etc.*), and reactive halogen species (RHS) (HOCl, HOBr, *etc.*)^[1]^. Neutrophil NADPH oxidase and myeloperoxidase (MPO) (EC 1.11.2.2), which catalyze the production of highly reactive ROS or RHS, respectively^[1–2]^, play a key role in the body's antimicrobial defense through various mechanisms that destroy pathogens. The roles of ROS and RHS in the formation of neutrophil extracellular traps (NETs), which are powerful and specific tools of neutrophils used to capture and kill viral, fungal, bacterial, and protozoal pathogens, have been actively studied^[3]^. NETs are extracellular fibrillary structures composed of decondensed DNA complexed with granule proteins (neutrophil elastase [NE], lactoferrin, MPO, cathepsin G, *etc.*), histones, and various cytoplasmic proteins^[4]^. These molecules can directly kill microorganisms or inhibit their growth by destroying virulence factors or disrupting the cell membrane. However, excessive NET formation is implicated in the development and complications of many diseases, including diabetes mellitus, thrombosis, autoimmune diseases, and viral diseases, among others^[5]^, conditions in which oxidative/halogenative stress and inflammation occur. Low-density lipoproteins oxidized by MPO-produced species have been shown to stimulate neutrophils, inducing NET formation and enhancing NET-mediated inflammatory responses in vascular endothelial cells, thereby contributing to the pathogenesis of cardiovascular diseases^[6]^. Chlorinated lipids that arise from MPO-dependent reactions also cause NET formation^[7]^. However, the mechanisms that regulate the MPO-related triggering of NETosis, the process of NET formation, are not yet fully understood.

It has been found that an increase in the concentrations of MPO and its products, which usually occurs at sites of acute inflammation, can lead to modifications of biomolecules^[8–9]^. Human serum albumin (HSA), which is abundant in the blood and interstitial fluid, and other plasma proteins can scavenge MPO-generated hypohalous acids, thereby eliminating these strong oxidizing agents. However, plasma proteins are no longer considered merely passive scavengers of hypohalous acids. Studies on the role of protein oxidation products in infectious and inflammatory processes indicate that these proteins exhibit both protective and pathological effects^[1,10]^. Exposure of proteins to MPO-derived oxidants, especially HOCl, results in a wide range of modifications. The primary targets for HOCl in proteins are Met, Cys, His, Trp, Lys, and Tyr residues^[11]^. Reactions with functional groups of these amino acid residues can change the physicochemical properties and conformation of the peptide chain^[12]^. Other investigators and our group have demonstrated that HOCl-modified HSA (HSA_HOCl_) causes various neutrophil responses, such as NADPH oxidase activation, degranulation, cell shape changes, and actin cytoskeleton reorganization^[13–15]^. HSA_HOCl_ has also been shown to prolong neutrophil survival in the presence of highly immunogenic foreign antigens by binding to these antigens and preventing their uptake by immune cells^[13]^. Inoue *et al*^[16]^ demonstrated that a disturbance in the plasma redox potential as a result of HSA thiol oxidation, which can be caused, among other things, by HOCl, promotes NETosis.

Thus, there is every reason to believe that HSA_HOCl_ acts as a proinflammatory mediator through various mechanisms. In the present study, we investigated whether HSA_HOCl_ could be a regulator of NETosis.

## Materials and methods

### Reagents

Sodium hypochlorite solution, HSA (Cat. #А1887), Histopaque-1077, phorbol-12-myristate-13-acetate (PMA), sodium citrate, trypan blue, 4-aminobenzoic acid hydrazide (4-ABAH), diphenyleneiodonium chloride (DPI), wortmannin, paraformaldehyde, and Triton X-100 were purchased from Sigma-Aldrich (St. Louis, MO, USA). Dextran T70 was obtained from Carl Roth GmbH & Co. KG (Cat. #9228.2, Karlsruhe, Germany). Aminophenyl fluorescein (APF; Cat. #131887), SYBR Green (Cat. #S7563), SYTOX Green (Cat. #1938594), Alexa Fluor 488-conjugated phalloidin (Cat. #1304569), Fluoromount mounting medium (Cat. #00-4958-02), and poly-L-lysine (Cat. #P8920) were from Invitrogen (Cambridge, UK). The anti-MPO monoclonal antibody was obtained by us as described previously^[17]^. Cyanine5 NHS ester was from Lumiprobe (Cat. #23020, Moscow, Russia). Salts for buffer solutions were acquired from Belmedpreparati (Minsk, Belarus) and Reahim (Moscow, Russia).

### Preparation of HSA_HOCl_ and monoclonal antibody against HSA_HOCl_

The HOCl modification of HSA was performed at room temperature (23 ℃) for 1.5–2 h with moderate stirring, as previously described^[14]^. HOCl (30 mmol/L) was added to HSA (0.3 mmol/L) in phosphate-buffered saline (PBS; 137 mmol/L NaCl, 8.7 mmol/L Na_2_HPO_4_, 1.5 mmol/L KH_2_PO_4_, and 2.7 mmol/L KCl with pH 7.4) in a molar ratio of 100∶1. To obtain monoclonal antibodies against HSA_HOCl_, we used Milstein and Köhler's technique^[18]^ with modifications. Clones that produced antibodies against HSA_HOCl_ while reacting negatively with unmodified HSA in culture medium were chosen for hybrid selection. The selected clone 1H2 (an IgM antibody class) was inoculated into pristane-sensitized mice (BALB/c × DBA F2 hybrid mice, Rappolovo nursery, Leningrad Region, Russia), and after two weeks, the ascites fluid of these mice was collected. IgM was purified by cryoprecipitation, followed by fractionation by gel filtration on a Sephacryl S-400 HR column equilibrated with PBS. The properties of the obtained antibody 1H2 mAb were verified using a solid-phase enzyme immunoassay with HSA_HOCl_ as described previously^[17]^.

### Neutrophil isolation

Human primary neutrophils were isolated from the venous blood of healthy donors as previously described^[19–20]^. Venous blood samples were obtained from the Republican Scientific and Practical Center of Transfusiology and Medical Biotechnology (Minsk, Belarus). The study was conducted in accordance with the Declaration of Helsinki and was approved by the Ethics Committee of the State Institution "Republican Scientific and Practical Center of Transfusiology and Medical Biotechnology", Minsk, Belarus (Protocol code No. 1, April 10, 2022). All participating subjects voluntarily signed an informed consent form.

Blood stabilized with 109 mmol/L sodium citrate (9:1, v/v) was mixed with 6% dextran T70 solution (5:1, v/v) and incubated at room temperature for 40 min to allow erythrocyte sedimentation. The remaining erythrocytes in leukocyte-enriched plasma were lysed by osmotic shock, and the leukocyte-enriched suspension was layered over Histopaque-1077 and centrifuged at room temperature at 450 *g* for 15 min. The precipitated cells were washed with PBS. The obtained cell suspension contained no less than 95% neutrophils with a viability of at least 95% as determined by the trypan blue test.

### Measurement of intracellular RHS production

Intracellular RHS production by isolated neutrophils was studied using flow cytometry with APF^[21–22]^. Neutrophils (1 × 10^6^ cells/mL) in PBS supplemented with 1 mmol/L СаCl_2_ and 0.5 mmol/L MgCl_2_ were incubated with APF (2.5 μmol/L) at room temperature for 10 min. HSA_HOCl_ at different concentrations or PMA (50 nmol/L) as a positive control were then added, and the incubation was continued at 37 ℃ for another 15 min. Next, the cell suspension was diluted fourfold in PBS supplemented with 1 mmol/L СаCl_2_ and 0.5 mmol/L MgCl_2_, and analyzed on a CytoFLEX flow cytometer (Beckman Coulter, Miami, FL, USA). Neutrophils were gated based on the ratio of forward scattered light to side scattered light. A 488 nm laser was used for APF excitation, and the fluorescence was collected in the fluorescein isothiocyanate (FITC) channel using a 525 (± 40) nm filter. Data analysis was performed using the CytExpert 2.4 software (Beckman Coulter). Both the percentage of APF-positive cells in the neutrophil population and the median APF fluorescence intensity in the APF-positive cell population were used as quantitative parameters to characterize intracellular RHS production.

### Detection of NET formation by isolated neutrophils

NETs formed by isolated neutrophils were detected using flow cytometry. Neutrophils (1 × 10^6^ cells/mL) suspended in PBS containing 1 mmol/L CaCl_2_ and 0.5 mmol/L MgCl_2_ were incubated with different HSA_HOCl_ concentrations (0.25–1 mg/mL) at 37 ℃ for 0.5–3 h, after which SYTOX Green (50 nmol/L) was added to the cells for 5 min^[19,23]^. Neutrophils incubated with PMA (50 nmol/L) at 37 ℃ for 1 h were used as a positive control. In a series of experiments, before the addition of HSA_HOCl_, neutrophils were incubated at 37 ℃ for 10 min with 20 μmol/L DPI (an NADPH oxidase inhibitor), 100 μmol/L 4-ABAH (an MPO inhibitor), 100 nmol/L wortmannin (a phosphatidylinositol 3-kinase [PI3K] inhibitor), or a monoclonal antibody (0.25 mg/mL) against HSA_HOCl_. After staining, the cells were analyzed using a CytoFLEX flow cytometer (Beckman Coulter). Neutrophils were gated based on the ratio of forward scatter to side scatter. SYTOX Green was excited with a 488 nm laser, and emission was collected using a 525 (± 40) nm filter in the FITC channel. Data analysis was performed using the CytExpert 2.4 software (Beckman Coulter). Both the percentage of dye-positive cells in the neutrophil population and the median fluorescence intensity of the dye-positive cell population were used as quantitative parameters to characterize NETosis.

### Detection of NET formation in *ex vivo* whole blood

We employed blood smears to detect NETs formed *ex vivo* in whole blood. The collected capillary blood was diluted 4∶1 (v/v) with a 3% aqueous solution of ethylenediaminetetraacetic acid (EDTA) as an anticoagulant. Blood samples were incubated at 37 ℃ for 1 and 3 h in the absence or presence of HSA_HOCl_ at 0.5 mg/mL. After incubation, standardized blood smears were prepared, fixed, and stained sequentially with the fluorescent DNA-binding dye SYBR Green and an anti-MPO monoclonal antibody conjugated with cyanine5 NHS ester (anti-MPO/Cy5). Imaging was performed using a Nikon ECLIPSE Ni-Е microscope (Nikon Corp., Tokyo, Japan).

### Visualization of the actin cytoskeleton

The actin component of the neutrophil cytoskeleton was visualized by laser confocal microscopy using a spectral-analytical system based on a Nanofinder scanning confocal microscope (Tokyo Instruments Inc., Tokyo, Japan). Neutrophils (3 × 10^6^ cells/mL) in PBS containing 1 mmol/L CaCl_2_ and 0.5 mmol/L MgCl_2_ were incubated with HSA_HOCl_ (0.5 mg/mL) in the absence or presence of a monoclonal antibody (0.25 mg/mL) against HSA_HOCl_ at 37 ℃ for 15 min, fixed with 4% paraformaldehyde (at room temperature for 10 min), washed to remove the fixative, and applied to glass coverslips precoated with poly-L-lysine. Coverslips with adhered neutrophils were incubated with 0.1% Triton X-100 (at room temperature for 5 min) to permeabilize the cell membrane and then with Alexa Fluor 488-conjugated phalloidin (0.165 μmol/L) at room temperature in the dark for 40 min. The coverslips were washed to remove excess fluorescent dye with PBS and distilled water and then mounted on slides using Fluoromount aqueous mounting medium. Alexa Fluor 488 fluorescence was detected using a FITC filter set (excitation at 488 nm; emission at 505–550 nm). Data analysis was performed using the NanoFinder Data Viewer system software (version 9.2.1.11).

### Statistical analysis

Results were presented as mean ± standard error. The minimum number of independent experiments performed for each experimental point was three. The significance of the difference between means was determined by Student's *t*-test, considering the differences significant at *P* < 0.05. Statistical data analysis was performed using Origin 7.0 software (OriginLab Corporation, Northampton, MA, USA).

## Results

### NETosis in isolated human primary neutrophils and *ex vivo* whole blood in the presence of HSA_HOCl _

We previously demonstrated that HSA_HOCl_ activated the respiratory burst and secretory degranulation in neutrophils^[14]^. In the present study, we further investigated whether HSA_HOCl_ induces NETosis.

Flow cytometry has gained popularity as a method for NET detection^[24]^. Masuda *et al*^[23]^ validated that a flow cytometry assay using staining with the plasma membrane-impermeable DNA-binding dye SYTOX Green was a reliable quantitative method for detecting NET formation in isolated human peripheral neutrophils exposed to the NET inducer PMA. Thus, we used this approach in our experiments.

**Figure 1:**
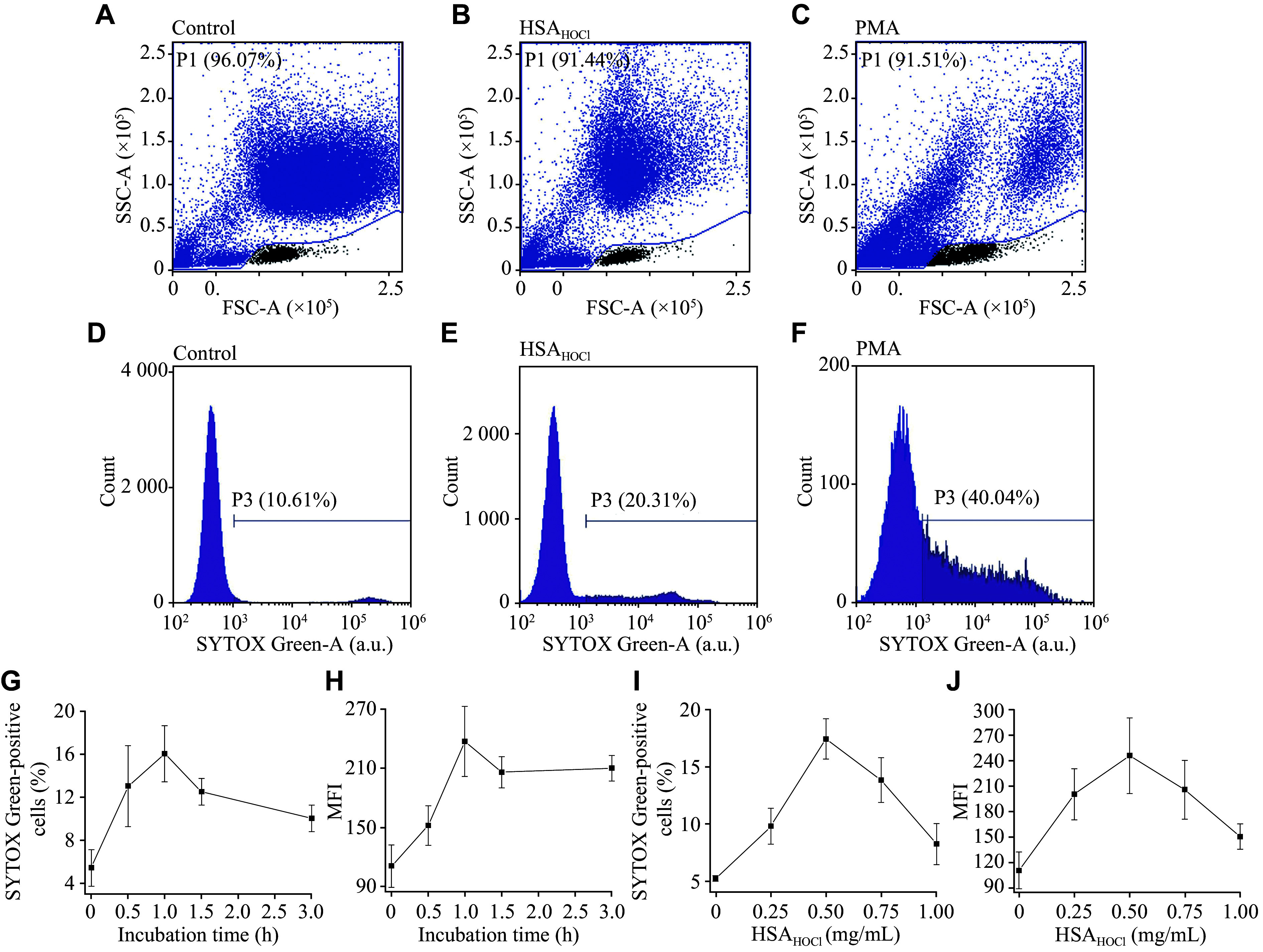
NET formation induced by HSA_HOCl_ in isolated neutrophils. NET formation was detected by flow cytometry using the DNA-binding fluorescent probe SYTOX Green. A–C: Forward/side scatter (FSC/SSC) dot plots showing changes in the size/shape of neutrophils after exposure to HSA_HOCl_ (B) or PMA (C), compared with control cells (A). D–F: Histograms of SYTOX Green fluorescence intensity for control cells (D), cells after exposure to HSA_HOCl_ (0.5 mg/mL, 37 ℃, 1 h) (E), and cells after exposure to PMA (50 nmol/L, 37 ℃, 1 h). The fluorescence with intensities from 10^3^ to 10^5^ arbitrary units (a.u.) reflects NETs. G and H: The HSA_HOCl_-induced NET production (at 0.5 mg/mL HSA_HOCl_) as a function of incubation time. I and J: The HSA_HOCl_-induced NET production (at 1 h of incubation) as a function of HSA_HOCl_ concentration. Dot plots and histograms are representative of three donors (*n* = 3). Data points on the graphs are mean ± standard error of the mean. Abbreviations: NET, neutrophil extracellular trap; FSC-A, forward scatter area; HSA, human serum albumin; HSA_HOCl_, HOCl-modified HSA; PMA, phorbol-12-myristate-13-acetate, a known inducer of NETs; MFI, median fluorescence intensity; SSC-A, side scatter area.

***Fig. 1A***–***1C*** present typical flow cytometric dot plots of SYTOX Green-stained neutrophils. PMA, a well-known potent inducer of NETosis, was included as a positive control and a reference for evaluating the potential of HSA_HOCl_ to induce NETs. The dot plots indicated that the exposure of neutrophils to PMA or HSA_HOCl_ led to changes in cell size/shape, likely indicative of NETs. The obtained histograms of SYTOX Green fluorescence intensity, representative examples of which are shown in ***Fig. 1D***–***1F***, indicated that approximately 10% of control neutrophils exhibited positive staining with SYTOX Green. The fluorescence intensity of these stained cells ranged from 10^5^ to 10^6^ arbitrary units (a.u.), suggesting that these cells were dead. The induction of NETosis by PMA led to the appearance of SYTOX Green-positive cells (approximately 40% of the total number of analyzed cells) emitting fluorescence with intensities of 10^3^–10^5^ a.u. After neutrophils were incubated with 0.5 mg/mL HSA_HOCl_ (the concentration with a strong activating effect on neutrophil respiratory burst and secretory degranulation^[14]^), SYTOX Green staining revealed a cell population (approximately 20% of all cells) with fluorescence between 10^3^ and 10^5^ a.u., apparently indicating NETs; meanwhile, the population of SYTOX Green-stained cells showed little fluorescence in the range of 10^5^–10^6^ a.u. Thus, HSA_HOCl_ appeared to induce NET formation, although it was a weaker inducer than PMA.

The extent of NETosis induced by HSA_HOCl_ was both time- and concentration-dependent (***Fig. 1G***–***1J***). The time course of NET production following neutrophil stimulation with 0.5 mg/mL HSA_HOCl_ showed that 1 h was the optimal incubation time to reach peak NET levels (***Fig. 1G*** and ***1H***). Over time, the number of SYTOX Green-positive events decreased, which could indicate NET degradation. NET formation increased in a dose-dependent manner, with a maximal stimulatory effect observed at 0.5 mg/mL HSA_HOCl_ (***Fig. 1I*** and ***1J***). Control flow cytometric experiments with native, unmodified HSA showed no effect on NET formation (data not shown).

**Figure 2:**
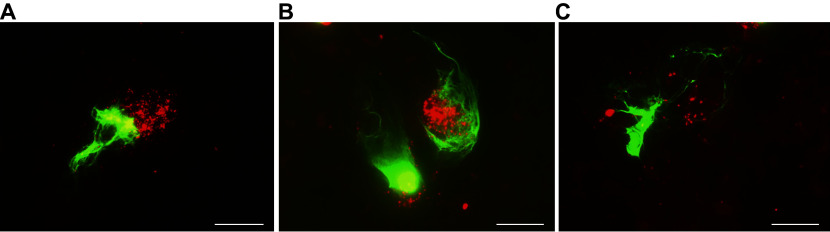
Representative images of NETs observed in *ex vivo* whole blood. Results obtained with fluorescence microscopy of blood smears stained for DNA and MPO using the DNA-binding dye SYBR Green and an anti-MPO monoclonal antibody conjugated with cyanine5 NHS ester (anti-MPO/Cy5), respectively. Green, SYBR Green; red, anti-MPO/Cy5. A: A recently formed NET. B and C: NETs undergoing degradation. Scale bar, 20 μm. Abbreviations: NET, neutrophil extracellular trap; MPO, myeloperoxidase.

NETs were also analyzed in *ex vivo* whole blood. To visualize NETs by fluorescence microscopy *via* co-localization of DNA and MPO, we stained blood smears with SYBR Green and a Cy5-conjugated anti-MPO monoclonal antibody (anti-MPO/Cy5)^[17]^. Notably, a number of NETs are consistently present in circulating blood. In both control blood and HSA_HOCl_-treated blood samples, NETs of different shapes and sizes were observed, with two NET types being particularly prominent. One type (referred to here as the first) was relatively small (approximately 20 μm in diameter) and compact (***Fig. 2A***). The other type (the second) was larger, forming a mesh-like network of thin DNA fibers (***Fig. 2B*** and ***2C***). It is reasonable to assume that the first type consisted of recently formed, not-yet-degraded NETs, while the second represented NETs that had already been circulating for a longer time and were undergoing gradual degradation (because of nucleases, proteases, *etc.*). Smears were made at 1 and 3 h of blood incubation with or without HSA_HOCl_ (0.5 mg/mL). Visual inspection of blood smears revealed that the presence of HSA_HOCl_ led to an increase in the number of the second type relative to the first type of NETs, which was especially marked at 3 h of incubation. These findings suggest that HSA_HOCl_ may accelerate NET degradation. Additionally, we observed NETs with voids within them (***Fig. 2C***), which may be considered an uncommon morphological variant of NETs.

### Participation of ROS/RHS and PI3Ks in the HSA_HOCl_-mediated NET formation

To elucidate the molecular mechanisms underlying the HSA_HOCl_-induced NETosis, inhibitor analysis was performed using DPI, 4-ABAH, and wortmannin. The results obtained when each of the inhibitors was added alone to neutrophils did not differ from those of control cells (data not shown).

**Figure 3:**
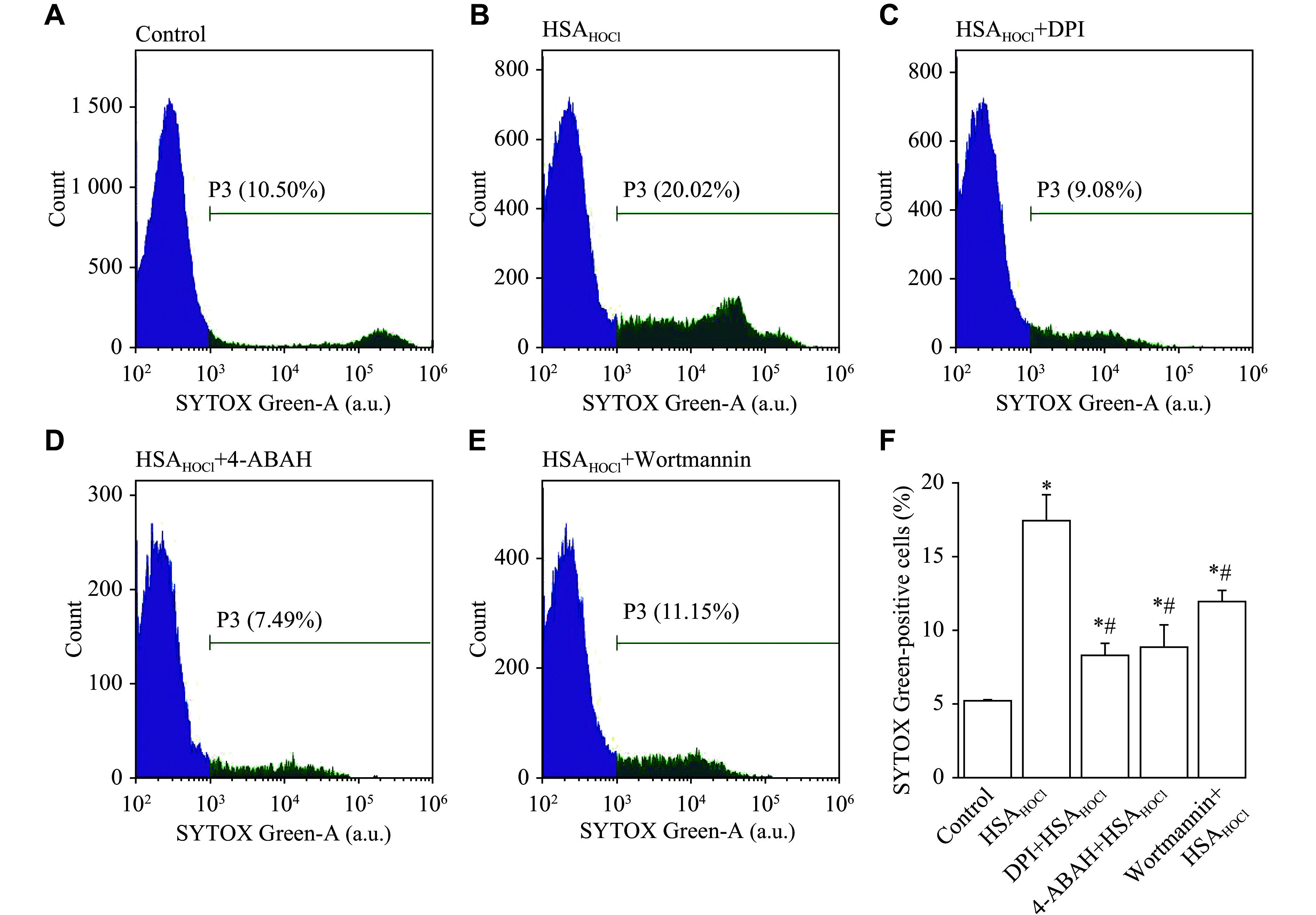
Effect of inhibitors of intracellular signaling systems on the HSA_HOCl_-induced NET formation. Neutrophils were pre-incubated at 37 ℃ for 10 min in the absence or presence of DPI (an inhibitor of NADPH oxidase; 20 μmol/L), 4-ABAH (an MPO inhibitor; 100 μmol/L), or wortmannin (an inhibitor of phosphatidylinositol 3-kinases; 100 nmol/L). HSA_HOCl_ (0.5 mg/mL) was then added to the test samples. Following 1-h incubation, SYTOX Green (50 nmol/L) was added to all samples and incubated for an additional 5 min in the dark. NET formation was detected by flow cytometry using the DNA-binding fluorescent probe SYTOX Green. A–E: Histograms of SYTOX Green fluorescence intensity, with intensities from 10^3^ to 10^5^ arbitrary units (a.u.) being recognized to reflect the formation of NETs. F: Bar graph representing sizes of the effect of HSA_HOCl_ on NET formation in the absence and presence of DPI, 4-ABAH, or wortmannin. Histograms are representative of three donors (*n* = 3). Bar values are mean ± standard error of the mean. *P*-values were determined by Student's *t*-test. ^*^P < 0.05 *vs.* the control group (neutrophils with no additions); ^#^*P* < 0.05 *vs.* the HSA_HOCl_ alone group. Abbreviations: NET, neutrophil extracellular trap; HSA, human serum albumin; HSA_HOCl_, HOCl-modified HSA; DPI, diphenyleneiodonium chloride; 4-ABAH, 4-aminobenzoic acid hydrazide; MPO, myeloperoxidase.

Because ROS are involved in the regulation of NET formation^[3]^ and we previously reported that HSA_HOCl_ stimulated neutrophil production of H_2_O_2_ and О_2_^•−[14]^, we investigated whether the HSA_HOCl_-induced NETosis was correlated with NADPH oxidase activity. For this purpose, we used DPI as an inhibitor of the NADPH oxidase assembly and activation, and found that neutrophil pre-incubation with DPI (20 μmol/L) inhibited the HSA_HOCl_-induced NETosis (***Fig. 3A***–***3C*** and ***3F***).

**Figure 4:**
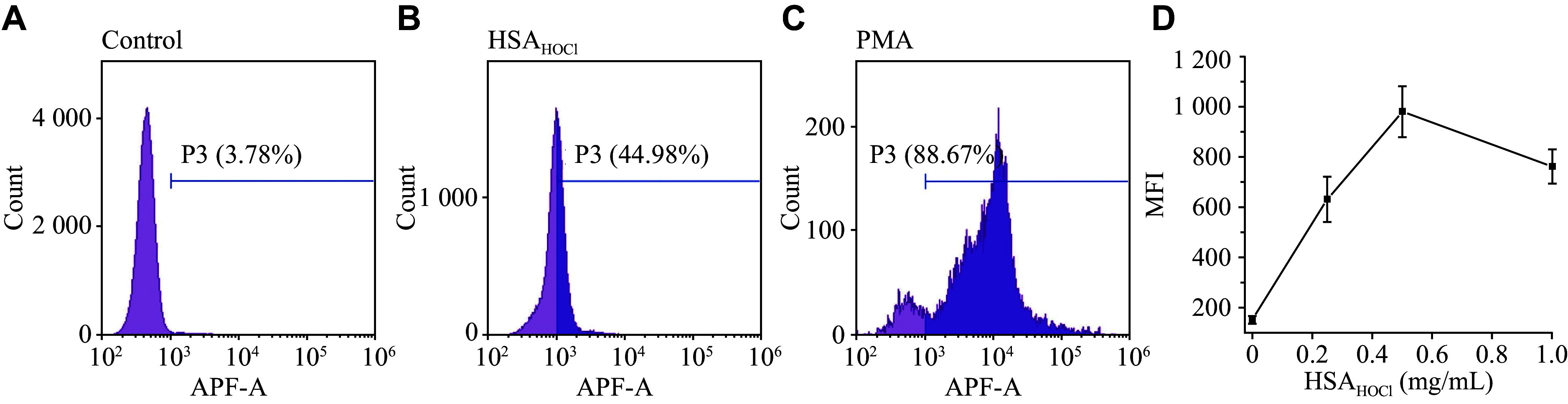
Flow cytometry analysis of APF-stained neutrophils treated by HSA_HOCl_ or РМА. A–C: Histograms of the APF (2.5 μmol/L) fluorescence intensity in control cells (A), cells incubated with HSA_HOCl_ (0.5 mg/mL, 37 ℃, 15 min) (B), and cells incubated with PMA (50 nmol/L, 37 ℃, 15 min) (C). D: Dependence on HSA_HOCl_ concentration for the median of fluorescence intensity (MFI) of APF in the APF-positive cell population. Histograms are representative of three donors (*n* = 3). Data points on the graph are mean ± standard error of the mean. Abbreviations: HSA, human serum albumin; HSA_HOCl_, HOCl-modified HSA; PMA, phorbol-12-myristate-13-acetate, a known inducer of NETs; APF, aminophenyl fluorescein, a fluorescent probe interacting readily with reactive oxygen species and reactive halogen species (RHS), with greater selectivity for RHS, especially HOCl.

NET formation is known to depend on MPO and the HOCl it produces^[25]^. The HSA_HOCl_-induced NETosis was suppressed when neutrophils were pretreated with 4-ABAH (an MPO inhibitor), indicating the involvement of MPO and HOCl in the NETosis process (***Fig. 3D*** and ***3F***). Additionally, flow cytometry analysis revealed an increase in APF fluorescence as a result of adding HSA_HOCl_ to APF-stained neutrophils (***Fig. 4***). APF is a fluorescein-derived probe that interacts with various ROS, RHS, and RNS, with more selectivity towards RHS, especially HOCl^[21–22]^. The APF fluorescence intensity increased with the increasing HSA_HOCl_ concentration, and reached a maximum at 0.5 mg/mL HSA_HOCl_. The inducing effect on neutrophil intracellular RHS production was weaker for HSA_HOCl_ than for PMA (***Fig. 4B*** and ***4C***), just as it was for the weak potency of HSA_HOCl_, compared with PMA, in inducing NETosis (***Fig. 1E*** and ***1F***).

PI3Ks are known to be key molecules involved in the regulation of intracellular signaling mechanisms leading to NET formation^[26]^. We found that in the presence of wortmannin (a nonspecific PI3K inhibitor), the HSA_HOCl_-induced NETosis was significantly reduced, suggesting the involvement of PI3Ks in the regulation of the HSA_HOCl_-mediated NET production (***Fig. 3E*** and ***3F***).

### Regulation of the HSA_HOCl_-induced NETosis by monoclonal antibody against HSA_HOCl_

**Figure 5:**
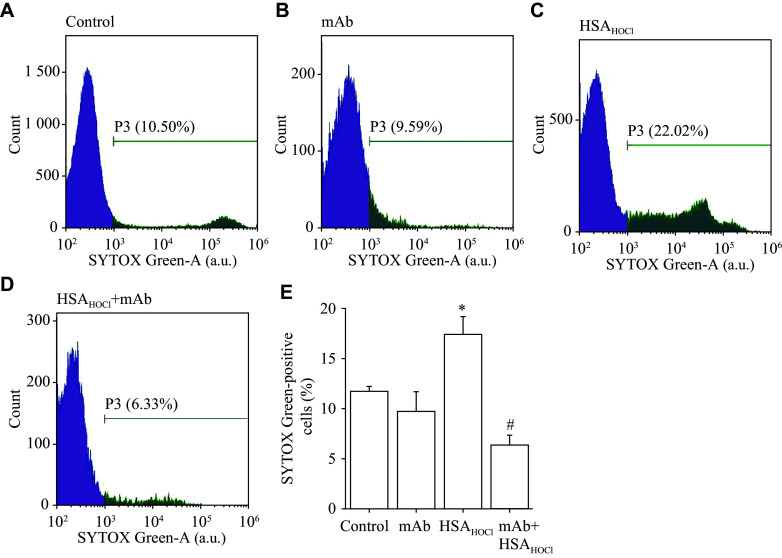
Inhibitory effect of a monoclonal antibody (mAb) against HSA_HOCl_ on the HSA_HOCl_-induced NET formation. NET formation was detected by flow cytometry using the DNA-binding fluorescent probe SYTOX Green. A–D: Histograms of SYTOX Green fluorescence intensity, with intensities from 10^3^ to 10^5^ arbitrary units (a.u.) being recognized to reflect the formation of NETs. E: Bar graph representing the sizes of the effect of HSA_HOCl_ on NET formation in the absence and presence of mAb. Neutrophils were incubated with HSA_HOCl_ (0.5 mg/mL), mAb (0.25 mg/mL), or their complex at 37 ℃ for 1 h. SYTOX Green (50 nmol/L) was then added and incubated for an additional 5 min in the dark. Histograms are representative of three donors (*n* = 3). Bar values are mean ± standard error of the mean. *P*-values were determined by Student's *t*-test. ^*^P < 0.05 *vs.* the control group (neutrophils with no additions); ^#^*P* < 0.05 *vs.* the HSA_HOCl_ alone group. Abbreviations: NET, neutrophil extracellular trap; HSA, human serum albumin; HSA_HOCl_, HOCl-modified HSA.

Based on Milstein and Köhler's hybridoma technique, we obtained a hybridoma clone, 1H2, that produces a monoclonal antibody against HSA_HOCl_ (1H2 mAb), and tested its ability to inhibit NET production by neutrophils exposed to HSA_HOCl_. The results showed that 1H2 mAb alone did not significantly affect the number of SYTOX Green-positive events or the number of NETs in the neutrophil suspension; moreover, HSA_HOCl_ complexed with 1H2 mAb failed to induce NETosis, indicating the ability of 1H2 mAb to block the neutrophil-stimulating activity of HSA_HOCl_ (***Fig. 5***).

**Figure 6:**
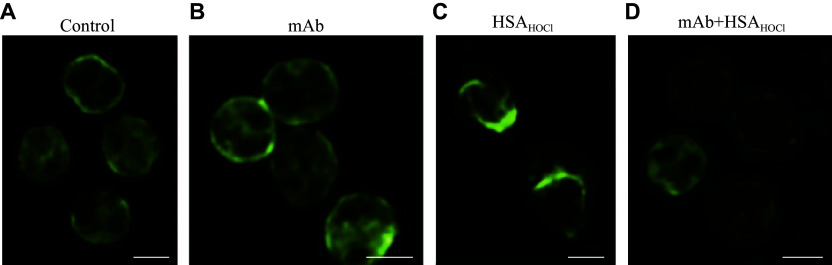
Neutrophil cytoskeletal rearrangement induced by HSA_HOCl_ and its prevention by a monoclonal antibody against HSA_HOCl_. A–D: Representative images of the distribution of Alexa Fluor 488-conjugated phalloidin, which binds to fibrillar actin, in control cells (A) and cells treated with an anti-HSA_HOCl_ monoclonal antibody (mAb) (B), HSA_HOCl_ (C), or their complex (D). Alexa Fluor 488 fluorescence was detected using excitation at 488 nm and emission at 505–550 nm. The concentrations were as follows: mAb, 0.25 mg/mL; HSA_HOCl_, 0.5 mg/mL; phalloidin, 0.165 μmol/L. Scale bar, 5 μm. Abbreviations: HSA, human serum albumin; HSA_HOCl_, HOCl-modified HSA.

Because cytoskeletal actin rearrangement is considered a requirement for NETosis^[27]^, and we previously found that incubation of neutrophils with HSA_HOCl_ led to actin cytoskeleton reorganization^[14]^, we next investigated whether the 1H2 mAb could prevent this effect. As shown in ***Fig. 6***, in control cells as well as in cells treated with 1H2 mAb, actin was evenly distributed, with a slight accumulation at the cell periphery. After neutrophils were exposed to HSA_HOCl_, fibrillar actin was concentrated along the periphery of the cell at the membrane edge, where outgrowths (pseudopodia) appeared. However, this effect of HSA_HOCl_ was eliminated when HSA_HOCl_ was complexed with 1H2 mAb.

## Discussion

The formation of NETs is one of the mechanisms of innate immunity, and is important for inducing an inflammatory response to infection. At the same time, excessive NET formation can promote thrombosis, diabetes mellitus, and other diseases (including autoimmune, viral, and infectious diseases)^[5]^. Therefore, the molecular mechanisms of NETosis regulation are of great interest and have been actively investigated.

In the present study, we identified HSA_HOCl_ as an inducer of NETosis. It is plausible to assume that HSA_HOCl_ can be formed in the interstitium of inflamed tissues, where local concentrations of HOCl generated by accumulated neutrophils can reach values as high as 25–50 mmol/L per hour^[28]^. The interstitial fluid HSA concentration varies widely, with maximum values of about 300 μmol/L^[29–30]^. Thus, the molar excess of HOCl over HSA can be high enough to result in the appearance of modified forms of HSA. The concentration of HSA_HOCl_ in our experiments was 0.5 mg/mL (7.5 μmol/L), which is quite realistic under pathophysiological conditions.

Neutrophil degranulation is a key step during NETosis. Neutrophil granule proteins (NE, cathepsin G, lactoferrin, MPO, *etc.*) regulate NET formation *via* various mechanisms. For example, NE migrates into the nucleus, promoting chromatin decondensation and nuclear envelope disruption^[31]^. Lactoferrin, by binding to negatively charged DNA, inhibits NET formation^[32]^, and this inhibitory property is maintained even after HOCl modification of lactoferrin^[19]^.

MPO, along with NE, is a key regulator of NETosis. Neutrophils from persons with complete MPO deficiency are unable to form NETs^[33]^. Several molecular mechanisms of NETosis regulation by MPO have been described, both dependent on and independent of its enzymatic activity. For example, partial MPO deficiency or pharmacological inhibition of MPO causes only a delay and reduction in NET formation^[33]^. Exogenous MPO added to MPO-deficient neutrophils does not "save" NET formation, suggesting that the latter requires MPO translocation to the appropriate subcellular compartment. MPO and NE secreted from azurophilic granules collectively enhance chromatin decondensation, leading to cell membrane rupture and NET release, with the ability of MPO being independent of its catalytic activity. On the other hand, MPO-derived RHS initiates lipid peroxidation, and also contributes, independently of NE activity, to chromatin decondensation, nucleus swelling, and subsequent release of nuclear contents^[34]^. The results of the present study indicate another mechanism by which MPO can regulate NETosis. This mechanism involves HSA modification by HOCl, a product of MPO catalytic activity, which converts HSA into an agonist for NET formation.

To date, two major types of NET release have been distinguished: suicidal NETosis, which leads to cell death, and vital NETosis, in which the neutrophil can survive and maintain structural integrity^[4-5]^. Suicidal NETosis is dependent on the activity of NADPH oxidase, NE, and MPO, and is initiated by stimuli such as PMA, IL-8, LPS, and others. Vital NETosis usually does not involve NADPH oxidase activation and is induced by certain bacteria, particularly *Escherichia coli* and *Staphylococcus aureus*, through pattern recognition receptors that recognize molecular patterns on microbes. Suicidal NETosis usually requires 3**–**4 h to complete, although some cells undergo more rapid NET formation, taking 30**–**60 min post-stimulation to complete. Vital NETosis occurs much faster (within 5**–**15 min)^[3–5]^.

In the present study, we used different approaches to assess NETosis of HSA_HOCl_-treated neutrophils. Flow cytometric results demonstrated that SYTOX Green-positive cells were detected in HSA_HOCl_-treated isolated neutrophils, with their number increasing depending on the exposure duration and HSA_HOCl_ concentration. The increase in the proportion of SYTOX Green-positive cells after HSA_HOCl_ treatment of neutrophils was inhibited by inhibitors of ROS/RHS production and PI3K signaling, key events in NET formation, thus confirming that cells undergoing NETosis were detected. Collectively, these findings provide strong indication that HSA_HOCl_ can initiate NETosis. Moreover, the present results have been strongly supported by our recent *in vivo* study, which showed an increase in MPO, HOCl-modified HSA, and NETs in the blood plasma of patients with type 2 diabetes mellitus aggravated by inflammation associated with necrotic lesions of the lower extremities^[35]^.

We previously showed that HSA_HOCl_ activated NADPH oxidase in an integrin-dependent manner through PI3K activation and actin cytoskeleton rearrangement, causing neutrophil degranulation with the release of MPO^[14]^. In the present study, we have demonstrated that an increase in intracellular RHS production accompanied the action of HSA_HOCl_ on neutrophils, as also occurs with PMA^[36]^. The MPO inhibitor 4-ABAH and the NADPH oxidase inhibitor DPI blocked DNA release by neutrophils in response to HSA_HOCl_. A higher NET production at 1 h of neutrophil incubation with HSA_HOCl_, and the dependence of the HSA_HOCl_-induced NET formation on ROS/RHS, suggest that HSA_HOCl_ triggers a suicidal, ROS-dependent type of NETosis. Given the inhibitory effect of a PI3K inhibitor, we propose that the HSA_HOCl_-induced NET formation follows the PI3K/AKT-ROS-dependent signaling pathway^[26]^.

The decrease in NETs following longer exposure of isolated neutrophils to HSA_HOCl_ indicates NET degradation. It is known that HSA, being a negatively charged molecule (pI = 4.7), is capable of binding to positively charged histones in NETs. HOCl modification of HSA increases its negative charge, at least partly due to chlorination of free amino groups, which results in the loss of their positive charge. It may be proposed that such an increase in the negative charge should enhance the binding of HSA_HOCl_ to histones and cause their displacement from NETs, thereby destabilizing the latter. HOCl modification of HSA can also promote its interaction with other cationic proteins embedded in NETs, such as NE, MPO, and lactoferrin, thus disturbing the NET structure. At the same time, NET proteolytic enzymes can cleave other NET proteins. MPO-produced ROS and RHS can oxidize or chlorinate protein amino groups carrying positive charges, decreasing the protein's electrostatic affinity for DNA. ROS and RHS are also known to cause DNA degradation. Thus, the action of NET components may lead to the dissociation of histones and other proteins from DNA. In addition, during neutrophil activation accompanied by suicidal NETosis, cytoplasmic proteases and oxidases may be secreted from the cells and be implicated in NET disintegration.

For neutrophils in *ex vivo* whole blood, blood DNases are also involved in NET degradation. It may be proposed that HSA_HOCl_-mediated dissociation of proteins from DNA increases DNA accessibility to DNase hydrolysis. In experiments with *ex vivo* blood, HSA_HOCl_ was found to accelerate NET degradation, as indicated by an increase in the ratio of degrading NETs to newly formed NETs. Features such as a larger size combined with a large-mesh network of thin DNA threads were considered morphological signs of the disassembly of the initial, smaller and compact NETs.

We found a dependence of the NET number on HSA_HOCl_ concentration, with a decrease in NETs at higher concentrations. As we have earlier shown^[14]^, HSA_HOCl_ activates neutrophils *via* binding to a receptor (β2-integrin). Binding saturation is marked by a cessation of the increase in NETs, as seen at an HSA_HOCl_ concentration of 0.5 mg/mL. However, further increasing the concentration of HSA_HOCl_ was accompanied by a decrease in NETs, likely because excess HSA_HOCl_ begins to degrade existing NETs, with the consequences described above.

In our earlier study, HSA_HOCl_ was found to induce neutrophil NADPH oxidase activation, which was reflected by ROS generation and MPO degranulation^[14]^. The present results obtained using inhibitors of NADPH oxidase and MPO suggest that the activation of NADPH oxidase and ROS/RHS production are components in the pathway leading to HSA_HOCl_-dependent induction of NET formation. Ulfig *et al*^[13]^ revealed that HOCl-treated HSA activated neutrophil-like cells and triggered the respiratory burst, as observed by an increased generation of ROS detected by lucigenin-dependent chemiluminescence. It cannot be ruled out that a stimulatory effect of HOCl-treated HSA on neutrophils, which was demonstrated in the above-mentioned study, is mediated by the same modification in HSA. HOCl may cause various modifications to proteins, including oxidation of Cys, Met, Trp, and His residues, chlorination of Arg, Tyr, and Lys side chains, carbonylation, and intra- and intermolecular dityrosine cross-linking, with most of these modifications being irreversible. Salavej *et al*^[37]^ showed that when HSA was treated with about a 30-fold molar excess of HOCl, the oxidation of Met and Trp residues dominated, with no chlorination of any residues detected. At a 50- and 100-fold molar excess, with the latter used by us, basic amino acid side chains were also involved in the HOCl-HSA interaction, generating chloramines^[10,13]^. N-chlorination in HSA in response to HOCl has gained special attention, because it is considered one of the main mechanisms contributing to the immunomodulatory and protective effects of the modified HSA^[13]^. HOCl-modified HSA (with a 50**–**100-fold molar excess of HOCl) has been shown to exhibit chaperone-like activity, acting as a highly effective holdase-like chaperone and thus protecting other proteins from HOCl-induced aggregation^[13]^, as well as to exert an inhibitory effect on antigen uptake by pro-inflammatory macrophages, likely interfering with scavenger receptor-mediated endocytosis of antigens by binding them and preventing their uptake by the cells^[10]^. These effects can be reversed by two approaches: (1) reduction of chloramines formed on HOCl-treated HSA using ascorbate, methionine, or other antioxidants; and (2) selective methylation to block free amino groups of lysine and nitrogen atoms of arginine residues in HSA before exposure to HOCl^[10,13]^. Thus, these findings support an N-chlorination-based mechanism as the one that plays a crucial role in the physiological properties of HOCl-modified HSA.

The identification of HSA_HOCl_, which can stimulate NET formation, is of particular interest, because HSA_HOCl_ could be targeted by antibodies to block its binding to cells. This represents a potential strategy for treating diseases associated with excessive NET formation during inflammation accompanied by elevated MPO activity. Monoclonal antibody therapeutics have several advantages over synthetic pharmacological agents, such as high selectivity, high affinity for the target antigen even when the antigen level is low, and a common biodegradation pathway. We first derived the 1H2 mAb against HSA_HOCl_ and found that it inhibited the HSA_HOCl_-induced NETosis. In addition, 1H2 mAb prevented the HSA_HOCl_-induced reorganization of the neutrophil actin cytoskeleton. Thus, 1H2 mAb may be used to control the HSA_HOCl_-induced inflammatory response.

Combining our data and those of others suggests that MPO, and particularly HSA_HOCl_ arising from the MPO-mediated reactions, may act as a regulator of NET formation at sites of inflammation, adding to the list of compounds capable of activating/inhibiting NETosis. This study also demonstrates that a monoclonal antibody against HSA_HOCl_ may be used to regulate neutrophil function under conditions of increased MPO activity.
